# Tracing the Status of Silica Fume in Cementitious Materials Subjected to Deterioration Mechanisms with Raman Microscope

**DOI:** 10.3390/ma15155195

**Published:** 2022-07-27

**Authors:** Yanfei Yue, Jingjing Wang, Yun Bai

**Affiliations:** 1 College of Materials Science and Engineering, Chongqing University, 174 Shazheng Street, Shapingba, Chongqing 400044, China; 2 CRANN and AMBER Research Centers, Trinity College Dublin, D02 PN40 Dublin, Ireland; JJWANG@tcd.ie; 3 Department of Civil, Environmental and Geomatic Engineering, University College London, Gower Street, London WC1E 6BT, UK; yun.bai@ucl.ac.uk

**Keywords:** cement, concrete, Raman microscope, safety, silica fume

## Abstract

The status and stability of the unreacted silica fume (SF) agglomerates existing in concrete structures subjected to various deterioration environments is largely unknown, but is a critical issue which could cause public concern. This work employed a Raman microscope, which combines the Raman spectroscopy with a light optical microscope, to characterize the phase assemblage in 6-month-old SF blended Portland cement (PC) pastes after 3-month exposure to simulated deterioration mechanisms (viz. carbonation, chloride attack, or sulfate attack), in order to illustrate the status of SF. Unhydrated SF phases, in terms of amorphous silica (Raman shift at about 350–540 cm^−1^), were identified in the SF blended paste samples after being exposed to carbonation and sulfate attack, indicating that there is a potential hazard to the living system, especially the structures undergoing long-term ‘interactions’ with a contiguous environment.

## 1. Introduction

Ultra-high performance (UHP) concrete increasingly attracts attention worldwide [1]. Incorporation of nanomaterials in concrete is increasingly being recognized as the most effective approach to developing high-performance, even super-high performance, concrete [2,3]. Although attempts were made in recent years to use nanomaterials, such as nano-SiO_2_, nano-clay, and carbon nanotubes [4,5,6] to improve the performance of concrete, traditionally, silica fume (SF) is considered as the most widely available material for formulating high-performance concrete (HPC) [7,8]. However, as an ultrafine substance with an average particle diameter about 150 nm, SF may also have the common issues facing nanomaterials, i.e., potential safety hazards to living systems, as its ultrafine particles could be easily absorbed through the skin, lungs, or digestive tract, causing health risks to the living systems [9,10]. It is reported that high agglomerates exist not only in the raw SF, but also remain in the hardened cement matrix even after long-term hydration [11,12]. To improve public’s confidence in nano-technology in future construction applications, the condition and the status of the unreacted SF, in particular, the SF agglomerates, need to be closely traced and understood. In a previous study, the authors already demonstrated that by combining Raman spectroscopy with a light optical microscope, both the composition and physical morphology of the SF can be successfully recognized [13]. In particular, the SF agglomerates were clearly identified and traced in the raw SF slurry as well as in the early and longer-term hydrated SF–PC blends. Whilst lots of interests were generated since the publication of the previous work, further studies were considered necessary before this technology could be applied to the real world. This is because during their service life, concrete structures are exposed to and interact with their surrounding environment, and deleterious substances, such as O_2_, CO_2_, Cl^−^, and SO_4_^2−^, can usually penetrate into concrete and consequently trigger some chemical reactions that can not only modify the microstructure of concrete, but also degrade certain hydration products [14,15,16]. Concerns therefore could be raised over whether these degradation processes could affect the stability of SF agglomerates and consequently impose any threats to the living systems.

Among various concrete deterioration mechanisms, carbonation, chloride attack/ingress, and sulfate attack are considered as the main culprits for the degradation of concrete. Their deterioration mechanisms are therefore briefly summarized in the context of their possible effects on the stability of SF agglomerates as follows:

- Carbonation, a deterioration mechanism of concrete, is a chemical reaction between atmospheric carbon dioxide (CO_2_) and cement hydration products. Whilst virtually almost all of the Ca-bearing hydration products [i.e., calcium silicate hydrate (C–S–H), calcium hydroxide (Ca(OH)_2_, CH), and various calcium aluminate or ferro-aluminate hydrates] can react with the CO_2_ to produce calcium carbonate (CaCO_3_), silica gel, hydrated aluminum, and iron oxides, the dominant reaction is the reaction between calcium hydroxide (CH) and CO_2_, which will convert CH into calcium carbonate [17,18]. Therefore, carbonation can reduce the pH of the concrete pore solution, which can subsequently trigger the corrosion of reinforcing bars. Additionally, owing to the increased volume from the formation of calcium carbonate, the microstructure of the cement matrix could be densified [19]. On the other hand, due to the carbonation shrinkage, it could also be possible that some microcracks could be formed [20]. All these could potentially affect the stability of the SF agglomerates.
- Sulfate attack occurs when sulfate salts (i.e., SO_4_^2−^) ingress into the cementitious materials and subsequently react with the hydrates/phases of the cement (e.g., calcium hydroxide, tricalcium aluminate hydrates, monosulfoaluminate, unreacted aluminate, or ferrite phase) to form gypsum or/and ettringite (AFt) [21,22]. The formation of gypsum and ettringite is generally considered to be harmful to the hardened cementitious materials. This is because the formation of ettringite is accompanied by local volume increase and subsequent pressure build-up to the surrounding matrix, leading to the cracking, spalling, and even destruction of cementitious materials [23]. Again, once the cracks are formed [24,25], it could promote further interactions between the cement matrix and surrounding environment. As a result, the stability of the SF agglomerates could be affected.
- Chloride ingress is another severe deterioration mechanism to the steel reinforced concrete, as it could cause the depassivation of the passive film on the steel surface. On the other hand, chloride ions can react with hydrated aluminate phases, yielding the so-called Friedel’s salt (3CaO Al_2_O_3_ CaCl_2_ 10H_2_O) [26,27,28]. As Friedel’s salt occupies more volume than aluminate phases, there is a pore refinement owing to the intrusion of chloride ions. More importantly, the consumption of aluminate hydrates could change the chemistry environment of the cement matrices, which can potentially affect the stability of the SF agglomerates within cementitious materials.

Apparently, the deterioration mechanisms not only can modify the microstructure of hardened cementitious matrices, but more importantly, could degrade certain cement hydrates, and thus change the chemistry environment in concrete. Therefore, concerns could be raised over the stability of unreacted SF agglomerates in SF blended cementitious systems, and hence the safety of the living systems exposed directly or indirectly to the concretes.

Unfortunately, up to date, there is not much research carried out on investigating the status of the SF agglomerates existing in concrete. It is still uncertain whether the deterioration processes would decrease the stability of the SF agglomerates and consequently cause any safety issues to the living systems. The current work, built upon the success of using a Raman microscope for tracing the status of silica fume in raw SF slurry and hydrated systems in our previous work [13], intends to (i) trace the status of the SF within cementitious materials subjected to different deterioration mechanisms and (ii) demonstrate the feasibility and working capability of Raman microscopy for identifying the SF in deteriorated cementitious materials. The testing protocol established in the previous work was again employed to investigate the condition of SF as well as the hydration and deterioration products of SF–PC blends subjected to carbonation, sulfate attack, and chloride ingress.

## 2. Materials and Methods

### 2.1. Materials

The as-received SF slurry, a dark-grey slurry supplied by Elkem (Hampshire, UK) with a water content of 50%, was used as the raw material in this study. Its chemical composition is shown in Table 1. The Portland cement (PC) used in this study was CEM I (in accordance with BS EN 197-1: 2011) supplied by Quinn Cement (Northern Ireland, UK), and its chemical composition is given in Table 1 as well.

### 2.2. Manufacture of SF Blended PC Paste Samples

The SF–PC pastes were obtained by replacing 8% of PC with SF by weight (based on the solid SF content in the SF slurry) at a water/binder (w/b) of 0.40. This w/b was used to achieve the workability in terms of the mini-slump value in the range of 55 ± 5 mm. The 8% replacement level was selected in this study because it is a typical level used to formulate high-performance concrete in practice.

The pastes were mixed using a Hobart planetary mixer. Water was first poured into the mixing bowl, followed by the binder materials (PC/SF). After mixing for 90 s at low speed, the mixer was stopped for 30 s during which all the paste adhering to the wall and bottom of the bow was removed by a scraper and placed into the middle of the bowl. The mixer was restarted and run at low speed for a further 90 s. The total mixing time was 3 min. This mixing regime was found to be able to ensure that the SF–PC blends can be properly mixed. Immediately after mixing, the pastes were cast and sealed into disposable plastic centrifugal tubes (Ø30 mm × 115 mm) to avoid loss of moisture. After 24 h of initial curing in the tube at 20 (±1) °C, the specimens were removed from the tube and then covered with a water-saturated hessian before being sealed in plastic sample bags and stored in a curing room at a constant temperature of 20 (±1) °C. The wet hessian was regularly checked and replaced once the moisture level was low in order to ensure the samples were fully saturated.

### 2.3. Deterioration Regimes

To simulate the situation occurring in a real application, the SF-blended PC pastes were matured at 20 (±1) °C for 6 months before being exposed to different deterioration regimes, as detailed below. In addition, to obtain the information under the most severe possible deterioration conditions, the SF blended PC pastes were first ground into the powder with the fineness of 63 µm at the end of 6 months and then subjected to different deterioration regimes for 3 months before conducting Raman microscopy analysis as specified in Section 2.4.

The deterioration regimes employed include carbonation, chloride ingress, and sulfate attack. The relevant standards [29,30] were consulted but with certain modifications in order to further accelerate the deterioration. The detailed condition of each deterioration regime is as follows:

- Carbonation: a modified carbonation chamber (LEEC, Nottingham, UK) was employed in the carbonation test. The chamber was set to maintain a constant temperature of 20 (±1) °C, a carbon dioxide (CO_2_) concentration of 5 (±0.5)%, and relative humidity (RH) of 60 (±5)%, based on a laboratory established regime [31].
- Chloride attack: the 165 g/L sodium chloride (NaCl) solution as specified in the NT BUILD 443 [29] was used as the aggressive solution for the chloride ingress test. The 63 µm powders were placed at the bottom of the tank containing NaCl solution. The tank was then closed tightly and placed in the curing room at a constant temperature of 20 (±1) °C.
- Sulfate attack: the sodium sulfate (Na_2_SO_4_) solution, with a concentration of 50 g/L as specified in the ASTM C1012 [30], was used. Similar to the chloride attack, the 63 µm powders were immersed in the Na_2_SO_4_ solution for 3 months and then removed from the tank.

### 2.4. Raman Microscope Test

A Renishaw inVia micro-Raman spectroscopy system equipped with a charged coupled device (CCD) detector and a 488 nm (Ar^+^) wavelength laser source was employed to trace the status of SF in the current study. The data were processed using OriginPro 8.6 software (OriginLab Corporation, Northampton, MA, USA). This micro-Raman system combines the function of Raman spectroscopy and an optical microscope, in which the former can be applied to identify the phases existing in the hardened SF–PC powder, whilst the latter can be used to observe the morphology of various hydrated/deteriorated phases. During the experiment, a tiny amount of powder sample was put on a glass slide, which was then fixed on the test stage. The laser beam was focused onto the samples through an Olympus 100× magnification microscope objective, with the sampling level being controlled at about 0.7 mW in order to avoid any local heat effects on the dark grey SF samples. The Raman spectra were recorded with the established regime in our previous study [32], viz. exposure time of 10 s and accumulations of 10, in order to improve the signal-to-noise ratio (SNR). All the measurements were performed under a controlled temperature of 20 °C. For each sample, more than 3 locations were tested and compared, with only the typical results demonstrated in the current paper.

## 3. Results and Discussion

As aforementioned, in our previous study [13], the SF agglomerates in the raw SF slurry were clearly identified by a Raman microscope. Additionally, the SF agglomerates were also identified in both the early (22 h) and long-term (6-month) hydrated silica fume bearing Portland cement (SF–PC) pastes. In the current study, the feasibility of employing this Raman microscopy technique to trace the status of the SF agglomerates in the deteriorated SF–PC blends was investigated and the results obtained from the carbonation, chloride ingress, and sulfate attack are reported in the following sections, respectively.

### 3.1. Carbonation

**Figure 1:**
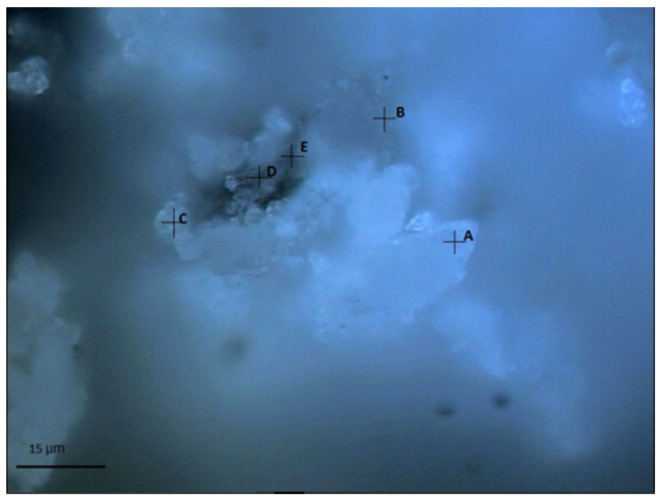
Light optical micrograph showing the selected testing area and points (A–E) in the carbonated SF–PC sample.

Similar to the procedures adopted in the previous work, the area with some interesting features was first identified by the optical microscope. The selected area was then characterized by Raman spectroscopy to reveal the compositions of the identified individual points in order to trace the location and the conditions of the SF. Figure 1 shows the light optical micrograph with the selected testing area and points in the carbonated SF–PC sample. As shown in Figure 1, a cluster area with interesting features was identified and selected with the optical microscope. In the cluster, a relatively dark core was noticed to be surrounded by a layer of white crystalline phases, with an ‘exit’-like point leaching out of these phases. To clearly identify the status of this cluster, five testing points at different locations were marked in the light optical micrograph (as shown in Figure 1) and analyzed by Raman spectroscopy. It can be seen that points A and C were located around the outside area, points D and E at the inner area, while point B was near the ‘exit’-like area.

**Figure 2:**
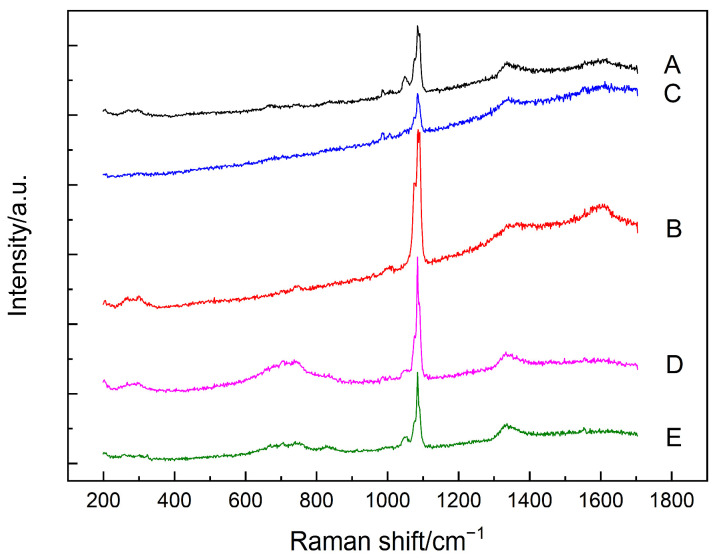
Raman spectra of the analyzed points (A–E) as shown in Figure 1.

Figure 2 presents the Raman spectra obtained at those five points selected in Figure 1. As expected, calcium carbonate was clearly identified in all the testing points, as evidenced by the most intense peak at 1085 cm^−1^ (CO_3_ symmetric stretching mode of calcite/aragonite), as well as the shoulders at 1074/1090 cm^−1^ (CO_3_ symmetric stretching mode of vaterite), probably due to the extensive carbonation reaction that occurred in the powdered samples under the accelerated carbonation condition [33,34,35]. Furthermore, the humps located around 1300–1400 cm^−1^ could be attributed to some unknown impurities within the samples. For points A, C, and B, the Raman spectra were also accompanied by a strong sloping background, together with two broad bands near 1360 cm^−1^ and 1605 cm^−1^ which could be from the carbon [36]. For points D and E instead, which were located at the inner area of the cluster, the hump displayed between 600 cm^−1^ and 800 cm^−1^ could be assigned to the Si–O–Si bending bands of C–S–H, which encompassed different degrees of silicate polymerizations, including Q^1^ dimers, Q^2^ chains, and Q^3^ units [37,38]. More importantly, silicon crystal and amorphous silica can be observed in point B, as indicated by the hump around 350–560 cm^−1^ (amorphous silica) and also by a peak at 517 cm^−1^ (crystal silicon) [39,40].

**Figure 3:**
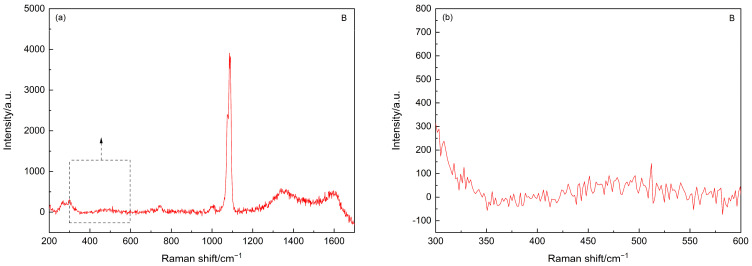
(**a**) Raman spectrum of the point B in Figure 1 after subtracting background. (**b**) Inset of Raman spectrum in (**a**) in the range of 300–600 cm^−1^.

As the main focus of the current study is to trace the status of SF agglomerates, the Raman spectrum of point B was further analyzed after subtracting the background, and the resultant spectra are shown in Figure 3. As shown in Figure 3b, after subtracting the background, the fingerprint information of the amorphous silica, i.e., the hump between 350 cm^−1^ and 560 cm^−1^ [40], can still be identified. Interestingly, the Raman spectra of these carbonated SF–PC paste samples showed some different features as compared to that of the 6-month-old SF–PC blends without being subjected to any deterioration environment as reported [13]. In the previous study, the hump related to the amorphous silica was observed at 400–530 cm^−1^, indicating the existence of unhydrated SF in the 6-months SF–PC blended paste. However, in the current study, the band of amorphous silica in this carbonated paste sample exhibited much broader features, with a nearly flat hump crossed between 350 cm^−1^ and 560 cm^−1^. This phenomenon could be due to the overlap of the Raman bands of the amorphous silica from both the residual SF and the silica gel, which might potentially formed from the decomposition of C–S–H gel under such a severe carbonation condition [18].

### 3.2. Chloride Attack

Chloride attack is another very serious deterioration mechanism, which can cause the corrosion of steel bars in reinforced concrete structures. The ‘total chloride’ ions can exist in concrete in two forms, i.e., ‘free chlorides’ in the pore solution and ‘bound chlorides’ by primarily forming Friedel’s salt. The former is the culprit responsible for the corrosion of steel bars, while the latter is invaluable for chemically immobilizing chloride ions, and hence can improve the durability of concrete structure. Therefore, in the current study, in addition to tracing the status of SF agglomerates in chloride attacked SF–PC blends, characterizing the Friedel’s salt could also have great practical importance. In a previous study [27], the authors, for the first time, successfully employed Raman spectroscopy in establishing the fingerprint information of the Friedel’s salt, with its full Raman spectrum (between 200 cm^−1^ and 4000 cm^−1^) being clearly revealed, including the featured Raman bands at 534/568 cm^−1^ and 783 cm^−1^ which correspond to the Al-OH stretching and bending vibration of Friedel’s salt. This information also extended to the current study for analyzing the Friedel’s salt formed in the SF–PC blends as detailed below.

**Figure 4:**
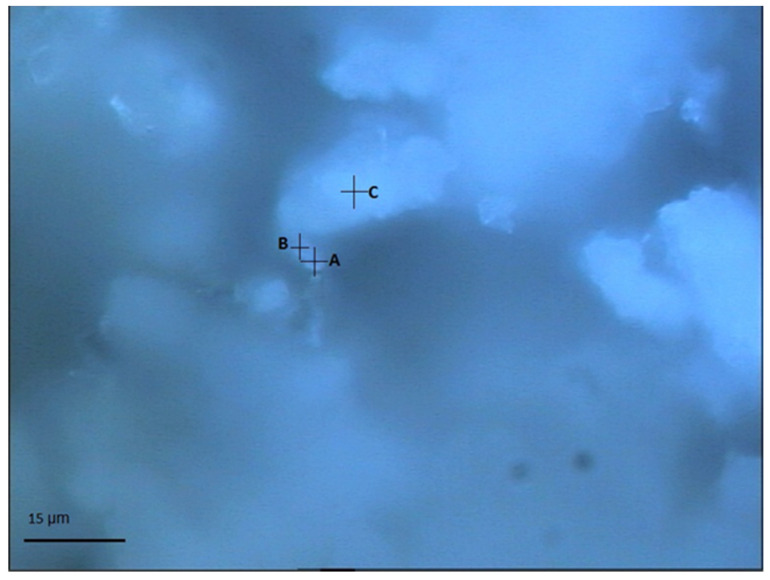
Light optical micrograph showing the selected testing area and points (A–C) in chloride attacked SF–PC sample.

**Figure 5:**
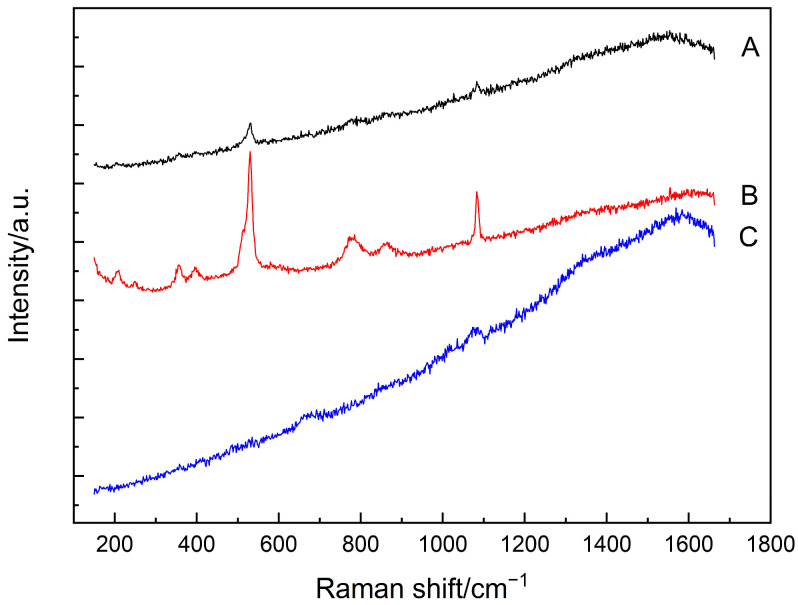
Raman spectra of the analyzed points (A–C) as shown in Figure 4.

Figure 4 presents the light optical micrograph showing the selected testing area and points to these chloride-attacked SF–PC blends. As shown in Figure 4, an area featured by a well-distributed bright region and dark grey zone was selected, and the related Raman analysis results are presented in Figure 5, accordingly. As can be seen from Figure 4, points A and B were located near each other, and the Raman analysis clearly identified some similar peaks in their spectra (as shown in Figure 5). The sharp peak at 513/530 cm^−1^ at points A and B can be assigned to the Al-OH stretching vibration of Friedel’s salt [27], whilst the peak observed at about 1084 cm^−1^ could be the CO_3_ symmetric stretching of calcite/aragonite formed [33,34]. Furthermore, compared to point A, more Raman bands of Friedel’s salt were recognized in point B, viz. 210/250 cm^−1^ (external rotation and translation), 356/395 cm^−1^ (Ca-O stretching vibration), 778 cm^−1^ (Al-OH bending vibration) [27]. At point C, C–S–H phases are identified, indicated by the hump at about 630–730 cm^−1^, which should correspond to the Si–O–Si bending bands of the silicate Q^1^ dimers and Q^2^ chains [37,38]. Evidently, no information about the unhydrated SF was identified from these three testing points based on the Raman analyses. One possible reason could be that, in addition to the sloping background, the overlapping of the main Raman peak of Friedel’s salt (513/530 cm^−1^) and the primary bands of silica (400–530 cm^−1^) could obstruct the differentiation of SF. This is because the Raman band of amorphous SiO_2_ is a weak hump, which could be hampered by the intense peak of Friedel’s salt. Hence, a testing point without Friedel’s salt needs to be selected for further study, viz, point C. Therefore, the background in the Raman spectrum of point C was subtracted to eliminate any possible disturbance from the background in distinguishing the weak hump of unhydrated SF.

**Figure 6:**
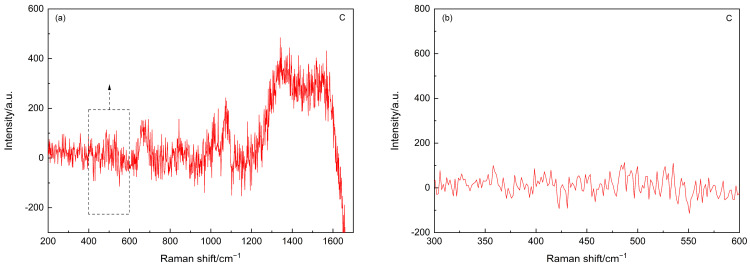
(**a**) Raman spectrum of the point C in Figure 4 after subtracting background. (**b**) Inset of Raman spectrum in (**a**) in the range of 300–600 cm^−1^.

Figure 6 presents the Raman spectra of point C after subtracting the background. However, as shown in Figure 6b, there is no Raman information about unhydrated silica being identified. In the literature, the chloride ions (Cl^−^), are considered to be able to accelerate cement hydration, probably owing to the following reasons:

- Cl^−^ ions, compared to OH^−^, have a relatively smaller ionic size and a greater tendency to diffuse inside the ‘membrane’ (e.g., adsorption/coating of Ca^2+^ on hydrated C_3_S), which could facilitate the build-up of internal pressure. This hence causes an early rupture of the ‘membrane’, leading to the unlocking of the C_3_S phase, which comes in contact with water and promotes the hydration reaction [41].
- The NaCl (used during deterioration) could react with CH in the pore solution and increase the amount of CaCl_2_ in the cement matrix. The CaCl_2_ is a well-established inorganic chloride-based accelerator and can flocculate hydrophilic colloids (e.g., C–S–H), facilitating the diffusion of ions and water through the initial C–S–H layer and thus allowing a higher rate of hydration during the early diffusion-controlled period [42].
- The CaCl_2_ could enhance the C–S–H nucleation by a homogeneous precipitation, and this accelerates the hydration [43].

Due to the accelerated hydration of the cement as elaborated above, the CH content could increase, which would, in turn, promote its reaction with SF, leading to the formation of additional C–S–H. As a result, compared to the SF–PC blends without being attacked by NaCl, more SF could be consumed due to the enhanced hydration of cement. This could explain the absence of the unreacted SF phases in this 3-month chloride attacked SF-PC paste sample.

### 3.3. Sulfate Attack

Sulfate attack, another severe deterioration mechanism, is an extremely complex phenomenon induced by the ingressive sulfate ions (SO_4_^2−^) through their reactions with cement hydrates/phases to form sulfate-bearing products, viz. the AFt from the reactions between SO_4_^2−^ and aluminate hydrates (e.g., tricalcium aluminate hydrates and monosulfoaluminate) or unreacted aluminate or ferrite phase, and the gypsum from SO_4_^2−^ reacts with calcium hydroxide (CH) [21].

**Figure 7:**
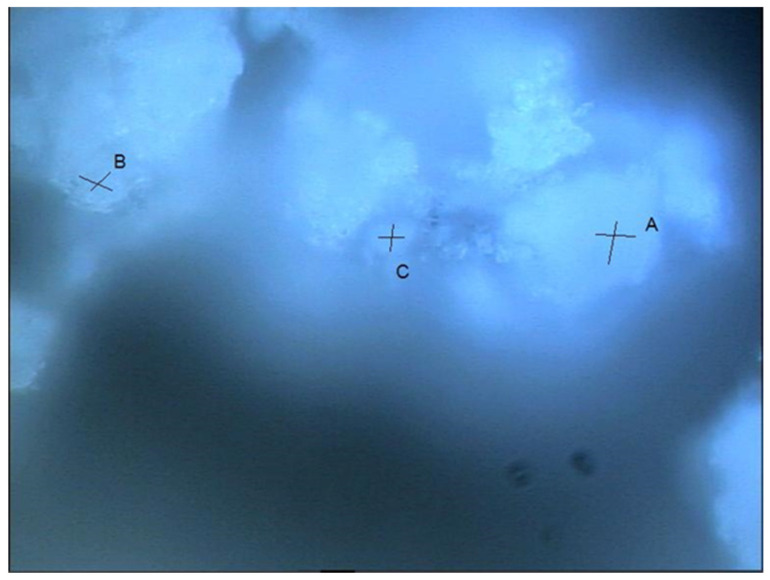
Light optical micrograph of selected testing area and points (A–C) from the sulfate-attacked SF–PC sample.

**Figure 8:**
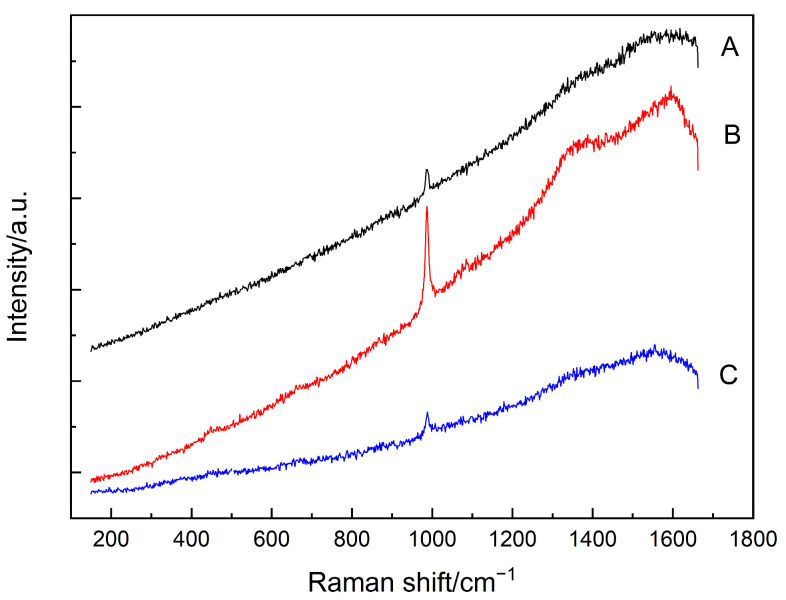
Raman spectra of the analyzed points (A–C) as shown in Figure 7.

**Figure 9:**
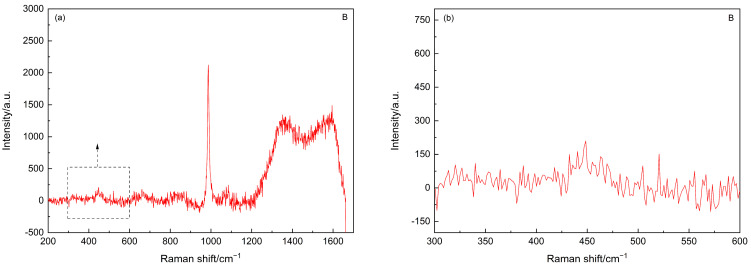
(**a**) Raman spectrum of the point B in Figure 7 after subtracting the background. (**b**) Inset of Raman spectrum in (**a**) in the range of 300–600 cm^−1^.

Figure 7 shows the selected testing area and points in this sulfate-attacked SF–PC sample, and Figure 8 presents the Raman spectra obtained from these 3 testing points. As can be seen from Figure 7, an area with a dark cover layer and light core was selected. From Figure 8, sulfate-bearing products were identified in all the three points by Raman spectroscopy, indicated by the peak at 989 cm^−1^, which can be assigned to the SO_4_ symmetric stretching band of ettringite [44]. Carbon still existed in the sample, as evidenced by the bands at 1356 cm^−1^ and 1603 cm^−1^, and these could be attributed to the carbon in the raw SF [36] since carbon is used to reduce quartz to manufacture silicon. At point B, the hump around 420–480 cm^−1^ could be attributed to the Si–O–Si symmetric bending of Q^4^ units in C–S–H phases [37,38]. However, further analyses were hampered by the strong sloping background in the spectrum. To clearly illustrate the various features in the Raman spectrum of Point B, the troublesome background was again subtracted and the resultant spectra are shown in Figure 9.

It can be observed from Figure 9a that a sharp peak located at 989 cm^−1^, which could be attributed to the SO_4_ symmetric stretching mode of ettringite [44] formed during the sulfate attack, is more evident than the peak in the original spectrum (i.e., Figure 8). Furthermore, as shown in Figure 9b, the amorphous silica is identified, as indicated by the hump lying at 400–540 cm^−1^ [40], which was similar to that in the hardened cement paste before subjected to sulfate attack (reported in our previous paper [13]). The amorphous silica could be from the residual SF, which indicates the existence of unhydrated SF agglomerates in the sulfate-attacked sample.

### 3.4. Discussion

To better understand the Raman fingerprints, which were identified under different deterioration mechanisms, relevant Raman bands and assignments are summarized and reported in Table 2. Apparently, amorphous silica existed in the SF–PC paste samples exposed to carbonation and sulfate attack, but is absent from the chloride attacked samples. It is worth noting that, in order to accelerate the deterioration, in the current study, the paste specimens were ground into 63 μm powder before being exposed to different deterioration conditions. In the case of carbonation, the powder samples were carbonated under a CO_2_ concentration of 5 (±0.5)% for 3 months. Under such a severe carbonation environment, the Ca-bearing cement hydration products (not only Ca(OH)_2_, but also C–S–H etc.) could react with CO_2_, leading to the decomposition of C–S–H and the formation of amorphous silica gel. This newly formed silica gel, along with those residual unhydrated SF, could be identified jointly by Raman microscope. Their Raman bands could overlap with each other, which can well explain the alteration of the Raman bands of silica from the 6-month SF–PC blends without being subjected to any deterioration environment (i.e., the hump at 400–530 cm^−1^) to that in the current study (i.e., a much broader feature with a nearly flat hump crossed between 350–560 cm^−1^). Under sulfate attack, the formation of ettringite could cause the degradation of cement hydrates (e.g., calcium aluminate hydrate) and subsequently the release of the residual unhydrated SF. On the other hand, as aforementioned, the chloride ions could accelerate the cement hydration and thus promote the formation of Ca(OH)_2_, which reacts and consumes the SF phases. Under this circumstance, SF has not been identified in the chloride attacked PC paste samples.

In the current study, the effects of different deterioration mechanisms on the status of SF agglomerates, as elaborated above, were confirmed experimentally using the unique fingerprint of Raman spectroscopy, i.e., the Raman band of amorphous silica at 350–540 cm^−1^. Additionally, as clearly shown in Table 2, the related deterioration mechanisms can also be nicely traced by Raman spectroscopy. These results suggest that there is a potential for developing the Raman-based technique into a sensor system for monitoring the health conditions of concrete structures in the future.

## 4. Conclusions

The current work employed the Raman microscope to identify the status and stability of the unreacted SF within SF–PC blends that were subjected to three different deterioration mechanisms, namely, carbonation, chloride ingress, and sulfate attack. The following conclusions can be drawn:

(1) The amorphous silica was identified in the SF–PC blends exposed to the carbonation and sulfate attack, as evidenced by the Raman bands at about 350–540 cm^−1^. No silica phases were identified in the chloride attacked SF–PC blends, which could be attributed to the enhanced hydration of cement and hence continued hydration of SF. These results indicate that there is a potential hazard to the living system, especially the long-term servicing structures exposed to a contiguous deterioration environment.
(2) This study clearly demonstrated the potential of employing the Raman microscope for tracing the status of silica fume in cementitious materials subjected to deterioration mechanisms, indicating that the use of Raman microscopes could be an effective approach to monitoring the status of nanomaterials, such as SF, in concrete structures.

## Figures and Tables

**Table 1:** Chemical composition of Portland cement and silica fume.

Oxides/%	SiO_2_	Al_2_O_3_	Fe_2_O_3_	CaO	MgO	K_2_O	Na_2_O	SO_3_
PC	23.00	6.15	2.95	61.30	1.80	0.68	0.22	2.50
SF	93.00	0.70	1.20	0.30	1.20	1.80	1.50	0.30

**Table 2:** Raman bands and assignments of SF–PC paste samples under different deterioration mechanisms.

Deterioration Mechanisms	Raman Bands (cm^−1^)	Assignments
Carbonation	350–560	amorphous silica
	1360/1605	carbon
	517	crystal silicon
	600–800	C–S–H
	1085	calcite/aragonite
	1074/1090	vaterite
Chloride attack	210/250, 356/395, 513/530, 778	Friedel’s salt
	630–730	C–S–H
	1084	calcite/aragonite
Sulfate attack	400–540	amorphous silica
	1356/1603	carbon
	420–480	C–S–H
	989	ettringite

## Data Availability

Data are contained within the article.
